# Hyoscyamia in Insanity

**Published:** 1880-09

**Authors:** 


HYOSCYAMIA IN INSANITY.

DR. JOHN P. GRAY, Superintendent of the New York State Lunatic Asylum, says: As a sleep-inducing remedy hyoscyamia will often succeed in cases of furious insanity, where other remedies fail, and it has the advantage that it can be easily and safely administered hypodermically. In some cases of violent mania, where there is failure in cerebral energy, a combination of hyoscyamia and morphia is desirable. I have given, in cases of depression bordering on melancholia, and cases of high nervous excitement with muscular restlessness, the following: ℞ Ext. nucis vom.; morph. brom. āā grs. 8; piperin grs. 10; hyoscyamia grs. 3. Ft. pil. 30. Sig. One twice a day, and reduce to one at night.

I have seen very beneficial results in this class of cases from the twentieth to the fiftieth of a grain of hyoscyamia three times a day. The dose of the crystal varies from the fiftieth to the half of a grain, and as high as three-quarters has been given.

There are some who may take large doses without any apparent effect. It may be fairly stated, I think, that if, after the administration of a few doses, it does not produce a quieting and calming influence, it should be discontinued, and other remedies, such as chloral, morphia, conium, or the bromides substituted. No remedy is universally applicable nor universally beneficial. In high excitement, where there is considerable plethora, I have found it advantageous to give the bromides internally and the hyoscyamia hypodermically, and in others to alternate hyoscyamia with the bromides. These remedies together are especially useful in mania associated with epilepsy.

In paroxysms in chronic insanity, where persons are in a state of mental perturbation, and under the control of marked delusions, and inclined to destroy or take off their clothing, and keep up what might be called a constant "fussing and mussing," small doses of hyoscyamia, internally or hypodermically, are very useful. This condition occurs in cases of incomplete dementia as well as in chronic mania. The medicine seems to relieve the muscular and nervous restlessness, and to quiet the cerebral perturbation.

We had long been familiar with the value of the other preparations of hyoscyamus in these cases, but the alkaloid is so much more active, and so much quicker in action, and gives such immediate relief to the irritability of the brain, that its value is conspicuous. As a rule in such cases it is not necessary to continue the remedy for any length of time. Indeed it is generally quite sufficient to give it once or twice a day, or once a day and once at night for a few days—then intermit. We have found it very useful as a medicine, and in no instance harmful. Discriminately used, it certainly aids in the comfort and restoration of the patient. To be able to give even reasonable brain-quiet to conditions of frenzy is quite as comforting and aidful as to relieve the restlessness of a fever patient by a bath, and saves from just so much useless wear and tear.

I have found it beneficial in hysteria, and also in chorea. I have not had the opportunity of personally observing its influence in delirium tremens.

We have not found it particularly valuable in chronic insanity, where very marked delusions are quietly held; that is, when the insanity is fixed and there is no raving or frenzy, and when, if there is resistance to food, care, etc., it is due to a quiet determination to carry out this purpose.—*American Journal of Insanity.*

